# Breeding Young as a Survival Strategy during Earth’s Greatest Mass Extinction

**DOI:** 10.1038/srep24053

**Published:** 2016-04-05

**Authors:** Jennifer Botha-Brink, Daryl Codron, Adam K. Huttenlocker, Kenneth D. Angielczyk, Marcello Ruta

**Affiliations:** 1 Karoo Palaeontology, National Museum, Box 266, Bloemfontein, 9300, South Africa; 2 Department of Zoology and Entomology, University of the Free State, Bloemfontein, 9300, South Africa; 3 Florisbad Quaternary Research, National Museum, Box 266, Bloemfontein, 9300, South Africa; 4 Centre for Environmental Management, University of the Free State, Bloemfontein, 9300, South Africa; 5 Department of Biology, University of Utah, Salt Lake City, UT 84112, USA; 6 Natural History Museum of Utah, Salt Lake City, UT 84112, USA; 7 Integrative Research Center, Field Museum of Natural History, Chicago, IL 60605, USA; 8 School of Life Sciences, University of Lincoln, Lincoln LN6 7DL, UK

## Abstract

Studies of the effects of mass extinctions on ancient ecosystems have focused on changes in taxic diversity, morphological disparity, abundance, behaviour and resource availability as key determinants of group survival. Crucially, the contribution of life history traits to survival during terrestrial mass extinctions has not been investigated, despite the critical role of such traits for population viability. We use bone microstructure and body size data to investigate the palaeoecological implications of changes in life history strategies in the therapsid forerunners of mammals before and after the Permo-Triassic Mass Extinction (PTME), the most catastrophic crisis in Phanerozoic history. Our results are consistent with truncated development, shortened life expectancies, elevated mortality rates and higher extinction risks amongst post-extinction species. Various simulations of ecological dynamics indicate that an earlier onset of reproduction leading to shortened generation times could explain the persistence of therapsids in the unpredictable, resource-limited Early Triassic environments, and help explain observed body size distributions of some disaster taxa (e.g., *Lystrosaurus*). Our study accounts for differential survival in mammal ancestors after the PTME and provides a methodological framework for quantifying survival strategies in other vertebrates during major biotic crises.

Mass extinctions reshape biological communities as a result of extensive biodiversity losses over short time periods and novel selective pressures that either trigger secondary extinctions or alter tempo and mode of evolution^1^. The post-extinction recovery phases are as important as the mass extinctions themselves for understanding models of ecosystem regeneration and group diversification, particularly in the case of organisms that survive extinctions at low diversity before radiating extensively. The most catastrophic crisis in Phanerozoic history, the Permo-Triassic Mass Extinction (PTME), was characterised by a rapid decrease in global biodiversity, leading to a radical restructuring of ecosystems 251.9 Ma^2^.

Both marine and terrestrial communities showed reduced diversity immediately after the PTME, a likely consequence of primary productivity losses that caused secondary extinction cascades^1^,^3^,^4^. Additionally, the climate of the continental Triassic was characterised by less predictable rainfall regimes, increased aridity^5^,^6^ and elevated temperatures^7^,^8^. These unstable conditions^9^ persisted for some 5 million years, such that even the survivors of the PTME may have been at heightened risk of extinction throughout this interval. However, extinction levels were not uniform across all organisms. Among tetrapods, some disappeared completely (e.g. gorgonopsians, pareiasaurs)^6^, others survived at reduced diversity (e.g., procolophonoids, therocephalians, dicynodonts)^6^,^10^,^11^, and yet others (e.g. temnospondyls, diapsids, cynodonts)^12^,^13^,^14^ flourished. A ‘Lilliput effect’ indicates reduced survivorship of large-sized Permian tetrapods across the Permo-Triassic Boundary (PTB). For instance, both therocephalian and cynodont therapsids became smaller^15^. In the case of therocephalians, both size decrease within lineages and preferential survival of small taxa have been observed. Triassic *Lystrosaurus* species also showed evidence of a body size reduction^16^,^17^, but it is unclear whether these species approached smaller asymptotic sizes than Permian *Lystrosaurus* species or whether they did not survive long enough to attain sizes comparable to their Permian relatives.

High mortality rates are known to affect populations from unstable and resource-limited environments^18^. If changes in growth patterns occur within a lineage, particularly in the aftermath of a catastrophic event, then analyses of such patterns across various species may provide additional clues to differential responses to a crisis. Ultimately, a species’ potential to modify its life history strategies may be key to its survival^19^,^20^,^21^. To test this proposition, we examined growth patterns in representatives of all boundary-crossing therapsid clades from the South African Karoo Basin (dicynodonts, therocephalians, cynodonts), using data from the largest histological database of Permo-Triassic non-mammalian therapsids compiled to date. The clades in question are appropriate for our investigation because they occupy a wide range of ecological habitats, adaptive zones, trophic levels, and body sizes. Special consideration is given to the dicynodont *Lystrosaurus*, the most iconic of all PTME survivors. This very abundant genus (3000 + specimens in museum collections) dominated Early Triassic ecosystems worldwide for millions of years during the post-extinction recovery phase. It thus provides a sufficiently large sample for studying population structure and differential survival during mass extinctions.

Identification of different ontogenetic stages in the fossil record is problematic, both because different definitions of maturity apply (e.g., asymptotic size, sexual maturity) and because some osteological proxies for maturity (e.g., osteohistological characters, ossification sequences, secondary sexual characteristics, asymptotic size) do not always coincide^22^. Nevertheless, we can make use of potential asynchrony in putative osteological correlates of maturity to gain insights into how animals may change their growth patterns and life history strategies during extinction events.

We assessed life histories using bone tissue microstructure because bone tissues are known to reflect rates and rhythms of postnatal development in vertebrates^23^. Our histology sample comprises 34 taxa (103 specimens; 177 limb bones and three ribs) belonging to the boundary-crossing clades Dicynodontia, Therocephalia, and Cynodontia, as well as gorgonopsian therapsids (a non-boundary crossing clade), altogether spanning some 20 million years of therapsid evolution (Supplementary Appendix 1). To gain insights into the demographic structure of extinct populations before and after the PTME, we investigated body size distributions in therapsids (Supplementary Appendix 2) as a framework for interpreting relative abundances of different age classes (using basal skull length [BSL in mm] as a proxy for body size)^15^,^24^ and, ultimately, to infer differences in survivorship rates.

## Results and Discussion

Most taxa were characterised by early rapid growth, indicated by the presence of fast-growing fibrolamellar bone, with its highly vascularised woven-fibred matrix associated with primary osteons. Among smaller therapsid species, a more organized tissue (parallel-fibred bone) and decreased tissue vasculature indicate lower overall growth rates than in larger species (see Supplementary Text), a typical condition for tetrapods^23^. In each species, larger, ontogenetically older individuals were characterised by slower-forming bone tissues with reduced spacing between growth marks in the outer cortex. This indicates that growth rates decreased during ontogeny as animals approached somatic maturity and asymptotic size. Although the animal may not have ceased growing altogether, growth deceleration marks a departure from the juvenile stage (see Supplementary Appendix 1 for details). Such a growth rate shift usually coincides with the onset of reproductive maturity in extant tetrapods that show multi-year growth to large body size^25^,^26^,^27^,^28^,^29^, and this has been postulated for other extinct species, e.g. non-avian dinosaurs^30^,^31^,^32^.

Reproductive maturity can be reached before or after asymptotic size. In some small mammals (e.g. rodents) and birds, asymptotic size is reached so rapidly (often within the first year) that reproductive maturity generally occurs after somatic maturity. In large reptiles and many medium to large-sized mammals, a decrease in vascularisation, decreased spacing between growth marks and/or deposition of slow growing lamellar or parallel-fibred bone in the outer cortex typically coincide with the onset of reproductive maturity^25^,^27^,^28^,^29^,^32^, although reproductive maturity may occur prior to this transition in larger species^27^.

In the therapsids analysed in this study, the transition from rapidly forming fibrolamellar bone to parallel-fibred or lamellar bone, a decrease in vascularisation towards the periphery, decreased spacing between growth marks and/or the appearance of outer circumferential lamellae (OCL) were used as indicators that an individual had reached reproductive maturity. Analysis of these features in individual fossils compared against % BSL_max_ (maximum basal skull length) showed that they generally appeared in Permian taxa by the time individuals had reached 70% BSL_max_. Growth marks indicate either periodic decreases (annuli) or pauses (lines of arrested growth or ‘LAGs’) in growth, which occur annually or seasonally^25^. Thus, growth marks observed prior to the onset of the slower-forming tissues indicated that it took several years for individuals to reach somatic maturity, and this pattern was widespread among Permian taxa. Growth marks in Triassic theriodonts and dicynodonts were generally absent or rarely present in the form of one or two annuli. There is no indication that skeletal maturity was reached in any of the Triassic *Lystrosaurus* specimens (i.e., all sampled individuals were still growing rapidly at the time of death, regardless of body size). Morphological features such as muscle scars, degree of ossification and the appearance of skull ornamentation are variable relative to body size among *Lystrosaurus* individuals^16^, thus osteohistology is likely the most reliable indicator of the ontogenetic stage of these specimens.

**Figure 1:**
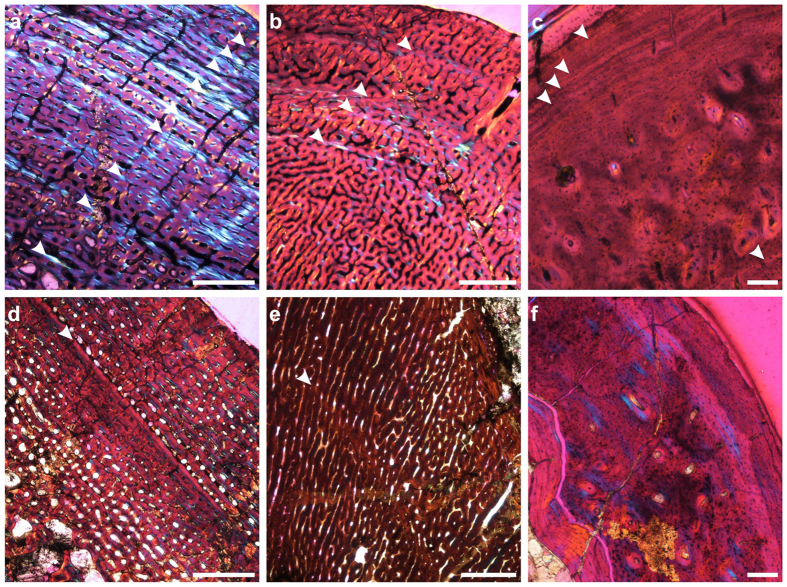
Osteohistological sections of Permian (**a–c**) and Triassic (**d–f**) late subadult or adult therapsids. Numerous growth marks (arrows) characterise Permian taxa, whereas two, but generally no growth marks characterise Early Triassic taxa. (**a**) Dicynodont *Lystrosaurus maccaigi,* humerus NMQR 3663a. (**b**) Therocephalian *Moschorhinus,* humerus NMQR 3939a. (**c**) Cynodont *Procynosuchus,* radius BP/1/3747. (**d**) *Lystrosaurus murrayi,* humerus BP/1/3236. (**e**) *Moschorhinus,* humerus BP/1/4227a. (**f**) Cynodont *Thrinaxodon,* radius BP/1/4282a. Scale bars equal 1000 μm (**a,b,d**); 500 μm (**e**); 100 μm (**c,f**).

**Figure 2:**
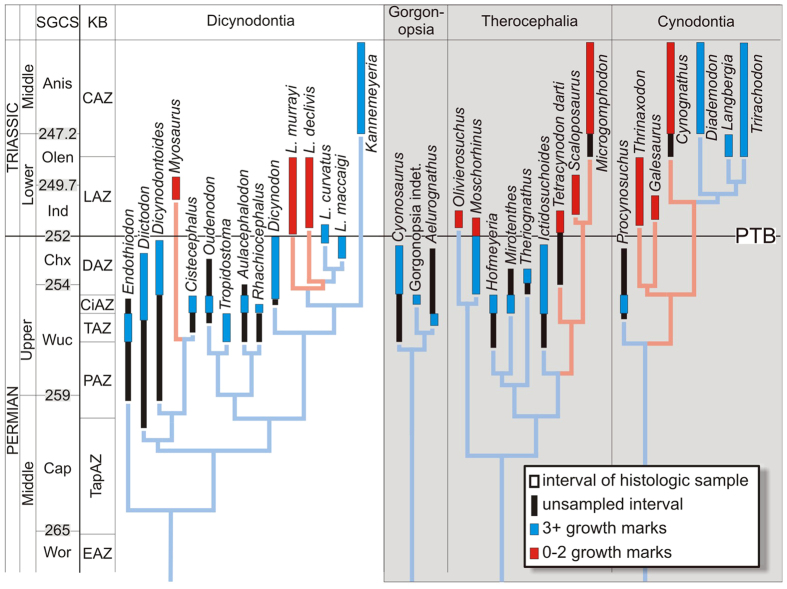
Growth mark counts mapped onto a phylogeny of Permo-Triassic therapsids. Coloured + black columns: observed stratigraphic ranges; faded colours: ghost lineages. Dates taken from^2^, phylogeny taken from^21^,^48^. Anis, Anisian; Cap, Capitanian; Chx, Changxingian; Ind, Induan; Olen, Olenekian; Wor, Wordian; Wuc, Wuchiapingian; CAZ, *Cynognathus* Assemblage Zone; CiAZ, *Cistecephalus* Assemblage Zone; DAZ, *Daptocephalus* Assemblage Zone; EAZ, Eodicynodon Assemblage Zone; LAZ, *Lystrosaurus* Assemblage Zone; PAZ, *Pristerognathus* Assemblage Zone; TAZ, *Tropidostoma* Assemblage Zone; TapAZ, *Tapinocephalus* Assemblage Zone; KB; Karoo Basin; PTB, Permo-Triassic boundary, SGCS, Standard Global Chronostratigraphic Scale. Grey shading indicates theriodont therapsids.

The most striking difference between Permian and Triassic therapsids is that the former had substantially more growth marks than the latter; this difference is significant even after accounting for inter-elemental variability (*p* < 0.0001; and see Supplementary Appendix 3), as well as phylogenetic relationships and body size differences amongst taxa (phylogenetic generalized least squares regression with mean number of growth marks as the dependent variable and BSL as a covariate; adjusted R^2^ = 0.4058; Fig. 1, Supplementary Figs 1–3; Supplementary Appendix 4 and 5). Permian therapsids also deposited growth marks at smaller relative sizes than Early Triassic therapsids, and went through prolonged, multi-year growth to reach adult body sizes (i.e., somatic maturity). In contrast, most Early Triassic therapsids revealed rapid sustained growth over fewer seasons (Fig. 2; Supplementary Figs 4 and 5).

Importantly, slower-forming parallel-fibred bone is absent from all Early Triassic *Lystrosaurus* specimens examined. Growth marks are rare: they are present in the largest specimens, but no more than one growth mark was observed. Even the largest known specimens of the Triassic species *L. murrayi* and *L. declivis* do not show any of the histological features typically associated with reproductive maturity (see Supplementary Fig. 4), such as decreased spacing between growth marks, a shift to slower-forming bone tissue, or the occurrence of outer circumferential lamellae. However, they do show evidence of a departure from the juvenile stage, in the form of an overall decreased vascularity compared to smaller individuals, and smaller, fewer vascular canals at the outermost peripheral cortex in places. Large specimens of *L. murrayi* and *L. declivis* are also very rare in museum collections worldwide, and this observation is unlikely to be due to a lack of preservation given the large number of Triassic specimens and the fact that large specimens tend to have a higher preservation potential in the fossil record. Based on these observations, we hypothesize that Triassic *Lystrosaurus* had shorter life expectancies and likely reached reproductive maturity at younger ages than Permian members of the genus, and before asymptotic size was attained (as suggested for some non-avian dinosaurs and many extant large-bodied reptiles)^30^,^31^. Small Triassic therocephalians and cynodonts do show evidence of approaching somatic maturity within few seasons^21^,^33^.

**Figure 3:**
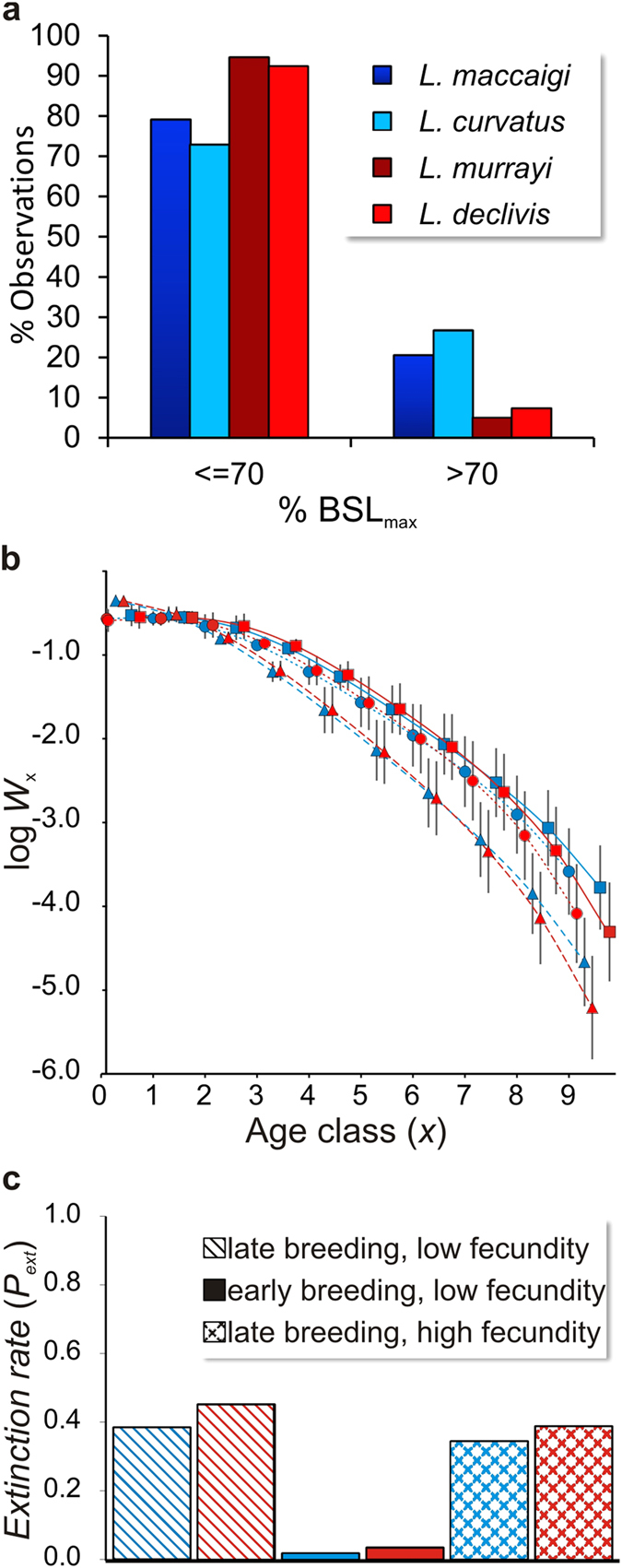
Differences in population structure, life expectancy, age at reproductive maturity and fecundity. (**a**) Body size distributions based on basal skull length (%BSL_max_) showing distinct differences between Permian (blue) and Triassic (red) species of *Lystrosaurus* (see text and Supplementary Material for results of chi-squared analysis comparing frequency distributions of larger and smaller individuals across taxa). (**b**) Modelled size class distribution under six scenarios: Early breeding (triangles) results in the observed Triassic pattern (i.e. fewer % of larger individuals). Blue, long life; Red, short life. Circles, late breeding and low fecundity; Triangles, early breeding, low fecundity; Squares, late breeding, high fecundity. Results are simulated stable size class distributions resulting from matrix model projections, presented as means with 95% confidence intervals over 1 000 permutations of each model condition. (**c**) Extinction rates for the six scenarios. Early-breeding taxa have the lowest extinction rates. log *W*_*x*_, proportion of individuals at size class X at stable size distributions; *P*_ext_, probability of extinction.

In order to test this hypothesis, and to understand more fully how differential growth influenced species’ apparent demographics and ecology, we compared body size distributions in *Lystrosaurus* before (*L. maccaigi, L. curvatus*) and after (*L. murrayi, L. declivis*) the PTME, using BSL data for 246 individuals (Supplementary Appendix 2; note that although *L. curvatus* did survive the extinction, representative specimens are rare [n = 2], and we excluded this small sample from the Triassic group). Because age and size are related, we expected post-extinction *Lystrosaurus* to have relatively fewer large individuals, as histological data suggest these taxa tended to die at earlier life stages. Consistent with this prediction, frequency-size distributions of the two Permian species differed from those of the Triassic species, in that the former distributions were normal (Shapiro-Wilks *p* = 0.498 and 0.152, respectively), whereas the latter were right-skewed, indicative of a bias towards smaller individuals (*p* < 0.01 and < 0.001, respectively; see Supplementary Fig. 6). Moreover, both Triassic species were represented by significantly fewer large individuals than were their Permian counterparts (i.e., >70% BSL_max_; *X*^2^ = 4.023 to 10.879, *df* = 1, *p* = 0.001 to 0.045; Fig. 3a). We also collected BSL data for a further 14 Permian dicynodonts, 13 of which had BSL distributions differing from those of Triassic *Lystrosaurus* (Supplementary Figs 6 and 7 and Supplementary Table S1; *Pelanomodon moschops* and *Dicynodon lacerticeps* were the exceptions). However, none of these taxa survived the PTME, so we could not examine within-lineage distribution shifts.

Results from both histologic and body size data are consistent with the hypothesis of a reduced life expectancy for Early Triassic therapsids. Taphonomic and collector effort biases are unlikely to have been responsible for the observed patterns, because such biases would affect primarily smaller individuals, and thus cannot explain the observation that relatively fewer larger individuals were recovered, when such individuals should in fact be more common. Moreover, although there was a change in taphonomic conditions between the Permian and Triassic, all tetrapod body sizes are documented in both periods, and relative fossil abundance does not decrease in the Early Triassic^17^, making taphonomic bias unlikely. Other large non-therapsid taxa are known from the same geologic horizons, including the predatory archosauromorph *Proterosuchus*. Thus, tetrapod fossils of all sizes are preserved in sedimentary facies, usually in mudrock (rarely in sandstone), and *Lystrosaurus* specimens are no exception. Especially noteworthy is the fact that collecting efforts have been evenly spread for the past 120 years across all Permian and Triassic stratigraphic intervals, encompassing a geographic area of some 730 000 km^2^. Intense sampling in all types of facies in the Early Triassic *Lystrosaurus* Assemblage Zone during the past 20 years makes collector and preservation bias highly unlikely^34^.

Due to the rarity of somatically mature therapsids in the Early Triassic (i.e. those in which bone histology displays a clear transition from higher juvenile growth rates to much slower growth over the course of ontogeny), and the abundance of *Lystrosaurus* during this time interval, we posited that reproductive maturity in these animals was likely reached before asymptotic size was achieved. Given that age at first reproduction was an unknown variable, we explored the implications of this hypothesis using simulations of population dynamics based on hypothetical life tables and size-structured matrix models (Supplementary Appendices 5–7). Simulations incorporated lower survival rates and higher environmental turbulence for Early Triassic populations. We predicted that, as Early Triassic species experienced shorter and less predictable periods suitable for growth, they would have modified their breeding strategies to compensate for lower survivorship rates. We considered two scenarios: (1) increased absolute reproductive output, implying larger clutch/litter sizes or more breeding events per year for the few individuals that reached large size; (2) reaching reproductive maturity earlier in life (Supplementary Fig. 8). Although these two scenarios are not mutually exclusive, we treat each of them in turn as their effects on population dynamics might be totally different, such that the potential benefits of each may vary across species with different body sizes, ontogenetic growth rates, etc. In our study sample, hypothesis (2) is consistent with our observations that Triassic therapsids appear to have died more frequently at younger ages than their Permian relatives, before they reached comparable body sizes.

Matrix model projections converged on stable size distributions that paralleled the observed demographic patterns in our BSL dataset: amongst earlier-breeding species, the largest individuals were comparatively under-represented (Fig. 3b). Moreover, our models revealed changes in extinction risk associated with each life history strategy. In turbulent environments, high population extinction rates were predicted (>40% of simulations; Fig. 3c and Supplementary Fig. 9). This figure was reduced to <3% by introducing an earlier onset of breeding, consistent with life history evidence for Triassic taxa. In contrast, elevated absolute breeding output did not appear to alleviate extinction risk. These results suggest that breeding earlier in life would have enabled larger-bodied therapsids such as *Lystrosaurus* or *Moschorhinus* to survive in turbulent, unpredictable environments that followed the PTME.

This study pioneers the concurrent use of bone histology, palaeoecological reconstruction, and ecological modelling to investigate the interplay amongst growth rates, life histories, and ecology, and their effects on differential survival amongst vertebrates during the PTME. It presents a generalized theory of survival strategy amongst therapsids that accounts for observed life history traits in the Triassic (size distributions and growth patterns), explains differential survival patterns, and meets predictions of ecological simulations. Because all of the data presented here was collected from Permo-Triassic therapsid fossils of the Karoo Basin, South Africa, future work should aim to compare changes in life history strategies in other basins and in other tetrapod groups (e.g., parareptiles, archosauromorphs).

In conclusion, the PTME was the most catastrophic of the five largest mass extinctions in Earth’s history, having produced a set of conditions that were unique to this event (e.g., extreme, prolonged instability in global carbon cycles)^35^. It has been suggested that the Early Triassic was a time of experimentation for life forms living in extreme conditions^36^,^37^. Our results show that Triassic therapsids generally did not attain large sizes, reached reproductive maturity (and sometime somatic maturity) within fewer seasons (supported by the presence of few or no growth marks prior to growth deceleration) and had shortened life expectancies. In contrast, their Permian relatives attained larger sizes and displayed prolonged, multi-year growth (evidenced by numerous growth marks) to somatic and reproductive maturity. As large Triassic *Lystrosaurus* individuals are absent, and collection/field observations suggest that this is not a sampling artefact, we propose that these individuals were likely breeding young to compensate for dying at an early age, a hypothesis supported by our modelling. Given the persistence and abundance of *Lystrosaurus* in the Early Triassic, we posit that they experimented with new life history strategies. *Lystrosaurus* may have had unusual developmental plasticity compared to Permian dicynodonts and may have been able to breed while still growing fairly rapidly. Our demographic simulations reveal how such a shift to breeding at younger ages in the face of reduced life expectancies could have helped therapsids survive the harsh, unpredictable environmental conditions of the Early Triassic, and that this change in life history played a critical role in allowing *Lystrosaurus* to become the most abundant terrestrial vertebrate during these turbulent times.

## Materials and Methods

### Osteohistology

Thin sections were prepared by JB-B and AKH using standard procedures^38^, analysed with a Nikon Eclipse 50i Polarizing microscope, a DS-Fi1 digital camera, and processed in the image analysis programs NIS Elements D 3.2 and Image J. Further image processing and preparation were undertaken in CorelDraw and Adobe Photoshop. The description of osteohistological features and terminology follow Francillon-Vieillot *et al*.^39^.

The count of growth marks (Supplementary Appendix 1), which comprise annuli or lines of arrested growth (LAGs), was based solely on the observation of the thin sections. Retrocalculations (i.e. estimates of the number of missing growth marks due to secondary remodelling of the inner, and presumably younger, cortex) were not undertaken as there is little consensus on which method is the most appropriate for such calculations. Furthermore, the presence of growth marks in Triassic taxa is sporadic and often non-existent, making retrocalculation ineffectual. Assuming a consistent pattern of growth mark deposition across taxa, the observation of three or more growth marks is taken to indicate multi-year growth to reproductive maturity. This was based on the observation that some yearling rodents may show up to two growth marks in their bone tissues as some cohorts may represent different reproductive events (winter, summer)^40^. Secondary remodelling is typical of larger taxa, particularly in dicynodonts. However, even discounting missing growth marks due to this process, Permian taxa still show several growth marks in their cortex prior to a decrease in growth rate. In the Triassic *Lystrosaurus* species, only a single annulus was observed in each of the largest individuals in this study (representing 100% BSL_max_ in *L. murrayi* and 82% BSL_max_ in *L. declivis*). A single annulus was present in a few smaller and presumably ontogenetically younger individuals. Superimposition of these smaller sections onto the largest sections of each species indicated that only one annulus had probably been resorbed by secondary remodelling in the largest, presumably ontogenetically oldest, individuals in this study (Supplementary Fig. 4).

### Demographics and Ecology of Permo-Triassic Therapsids

Frequency-size distributions of each taxon were tested for deviations from normality, i.e. whether they were right- or left-skewed, reflecting under-representation of large or small individuals, respectively, using Shapiro-Wilk’s tests. Comparisons between *Lystrosaurus* species, and between other taxa with Permian, Early Triassic, or Mid-Triassic assemblages, were made using Pearson’s chi-squared tests based on 2 × 2 contingency tables (with Yates correction in cases where the number of individuals within one or more cells was <5), with each group divided into numbers of larger (>70% BSL_max_) and smaller individuals (≤70% BSL_max_). This approach is based on observations of the Permian and Triassic taxa in this study where a decrease in growth rate is evident by approximately 70% BSL_max_ (apart from Triassic *Lystrosaurus*). Frequency distributions of ontogenetically older and younger individuals were calculated by expressing each individual as a percentage of the total BSL range estimated for a taxon, from


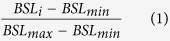


where BSL_i_ is the focal individual, and BSL_min_ and BSL_max_ reflect the minimum and maximum BSL values for the focal taxon. BSL_max_ is simply the largest BSL value observed for any taxon, but BSL_min_ had to be estimated because it is unlikely that the smallest individuals represented by each taxon have actually been recovered from the fossil record. Hence, we used allometric relationships between adult and neonate body sizes, such as are gleaned from extant vertebrates^41^, to estimate BSL_min_ for each taxon. Note that scaling exponents for these allometries differ significantly between viviparous (~0.9) and oviparous (~0.5 to 0.6) vertebrates^41^. However, we found no significant differences in resulting trends when either exponent was applied to our data, assuming that all taxa followed the same reproductive strategy. Therefore, we report only results based on a scaling exponent of 0.9. We acknowledge that more information about reproductive strategies in therapsids would surely improve the accuracy of our results. We anticipate that future applications of our approach are likely to answer key questions about reproductive strategies in other fossil vertebrate groups.

To determine the effects of different life history strategies on survival rates of Permian and Triassic vertebrates, we simulated ecological dynamics based on hypothetical size-structured life tables and matrix models that reflected differences in BSL distributions (Supplementary Appendix 6 and 7). Simulated life tables included 10 age/size classes (*x*) corresponding to 10 equally-sized BSL bins (0–10% BSL_max_, 10–20%, etc.): note this approach assumes linear growth, but due to the low number of growth marks observed in Triassic *Lystrosaurus*, we could not derive parameters for actual growth models. We also found no qualitative differences in analysis of BSL data using four (25% increments) or five (20% increments) size bins.

Life tables resembled either of two basic strategies, namely Type 1 (a convex relationship between survivorship and size/age) or Type 3 (concave) survivorships (see data in^42^), reflecting the extremes of a continuum observed in modern organisms generally. The former (Type 1) comprises organisms that generally produce few young, practice parental care, and suffer greatest mortalities later in life, whereas the latter (Type 3) comprises organisms that rely on very high reproductive outputs, allowing for high mortalities amongst juveniles, and low mortality rates amongst older, reproductive-age individuals. Survivorship schedules (*l*_*x*_) were calculated from age-specific survivorship schedules (*g*_*x*_) generated using the arbitrary formula


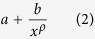


where *a* and *b* are positive and negative constants, respectively. The *g*_*x*_ schedules were then standardized to values between 0.05 and 0.9, and converted to *l*_*x*_ schedules by setting *l*_0_ = 1 and *l*_*x* + 1_ = *g*_*x*_*l*_*x*_. For negative values of *ρ*, equation 2 produces a concave *g*_*x*_, and convex *l*_*x*_, curve, i.e. reflecting Type 1 survivorships; for positive *ρ*, the *g*_*x*_ curve is positively asymptotic, and the *l*_*x*_ curve is concave, reflecting a Type 3 survivorship.

Fecundity schedules (*m*_*x*_) were also simulated so as to mirror trends observed in extant vertebrates, for which *m*_*x*_increases asymptotically with age, before falling after senescence (see data in^43^). We used the asymptotic formula


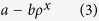


where *a* and *b* are constants >1, and 0 < *ρ* < 1, to simulate *m*_*x*_schedules. Subsequently, we simulated fertility schedules (*F*_*x*_), by standardizing *m*_*x*_ schedules to values between 1 and 3 for Type 1 populations, and between 1 and 20 for Type 3 populations (these maxima resemble realistic estimates for extant animals approaching the size of *Lystrosaurus* – see allometric parameters in^43^). Stochastic variation in *l*_*x*_, *g*_*x*_, and *F*_*x*_ was set at ±10% of the range for each parameter.

Simulations of population growth under a variety of life history, ecological, and environmental conditions, mimicking patterns observed in the histological and BSL data, were based on age-structured matrix models (Supplementary Appendix 7) derived from the above life tables (see e.g.^44^,^45^). Populations with higher survivorship rates and hence longer life expectancies were differentiated from shorter-lived ones by setting *ρ* in equation 2 to lower and higher values, respectively, whereby a larger *ρ* corresponds to a steeper slope and hence higher mortality rates especially amongst larger size/age classes (see Supplementary Table 2 and 3). We tested hypotheses relating to breeding strategy as follows: effects of early breeding, introduced by altering the minimum age (*x*) for which *F*_*x*_ > 0 was possible; and effects of enhanced reproductive rates introduced by increasing the maximum value for *F*_*x*_ to 5 and 30 for Type 1 and Type 3 populations, respectively. These two hypotheses are central to our idea that past populations with shortened life expectancies compensated for this shortfall by increasing reproductive outputs, attained either by reaching reproductive maturity at younger ages (or smaller size classes) or by increasing the absolute number of offspring (e.g. by having larger clutch sizes and/or more breeding bouts per year). Results are reported as means and 95% confidence intervals over 1 000 simulations for each set of conditions.

Finally, we explored population dynamics of populations using these various life history strategies under different environmental conditions. To this end, we used logistic growth models assuming direct density-dependence^46^, in which the equilibrium density (*K*) was taken to represent environmental carrying capacity. There are key differences between the concepts of equilibrium density and carrying capacity^47^, but for our purposes these variables may be assumed to capture similar aspects of population density dynamics. Two types of environments were simulated: a stable environment, in which *K* varied by ±10% across time intervals (*t*), and a variable environment in which *K* varied by ±50%, the latter reflecting the hypothesized stochastic and unpredictable Early Triassic environment. Growth rates (*r*) for these growth models were derived from the matrix models described above, where *r* = lambda −1. However, *g*_*x*_ schedules at each time interval were adjusted so as to reflect changes in *K*, using


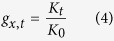


In other words, *g*_*x*_ at each time interval increased or decreased by a proportion equal to the proportionate change in *K*_*t*_ relative to the original *K.* Thus, growth rates in these models are dynamic, to a degree reflecting the instability of the environments. For each model variant, i.e. life history strategy, results reported here are means over 100 time intervals calculated from 1 000 iterations. Extinction rates are calculated as the percentage of simulations in which population sizes (*N*) reached <1 individual.

## Additional Information

**How to cite this article**: Botha-Brink, J. *et al*. Breeding Young as a Survival Strategy during Earth’s Greatest Mass Extinction. *Sci. Rep.* **6**, 24053; doi: 10.1038/srep24053 (2016).

## Supplementary Material

Supplementary Information

Supplementary Dataset 1
