# Supplementary material for: Breeding Young as a Survival Strategy during Earth’s Greatest Mass Extinction
Source: Sci Rep. 2016 Apr 5;6:24053. doi: 10.1038/srep24053 (PMC4820772; doi:10.1038/srep24053)
Supplement: Supplementary Information [file srep24053-s1.pdf]

Breeding Young as a Survival Strategy during Earth's Greatest Mass Extinction

Jennifer Botha-Brink[1,2], Daryl Codron[3,4], Adam K. Huttenlocker[5,6], Kenneth D. Angielczyk[7], Marcello Ruta[8]

[1]Karoo Palaeontology, National Museum, Box 266, Bloemfontein, 9300, South Africa.
[2]Department of Zoology and Entomology, University of the Free State, Bloemfontein, 9300, South Africa.
[3]Florisbad Quaternary Research, National Museum, Box 266, Bloemfontein, 9300, South Africa.
[4]Centre for Environmental Management, University of the Free State, Bloemfontein, 9300, South Africa.
[5]Department of Biology, University of Utah, Salt Lake City, UT 84112, USA.
[6]Natural History Museum of Utah, Salt Lake City, UT 84108, USA.
[7]Integrative Research Center, Field Museum of Natural History, Chicago, IL 60605, USA.
[8]School of Life Sciences, University of Lincoln, Lincoln LN6 7DL, UK.

Assignment of the PTB and the Permo-Triassic Transition in South Africa

The South African Karoo Basin preserves a detailed record of environmental and evolutionary changes associated with the PTME that has been thoroughly studied[1-10]. Currently, the PTB in the Karoo is defined biostratigraphically by the last appearance of *Daptocephalus leoniceps* and *Lystrosaurus maccaigi*[1,11]. The first appearance of *Proterosuchus fergusi* and *Lystrosaurus murrayi* delineates the earliest Triassic[1]. The Permo-Triassic transition in the Karoo is accompanied by an almost complete faunal turnover that has long been considered to represent the Permo-Triassic mass extinction[12]. Although precise radiometric dates are unavailable to tie this turnover to the marine GSSP, the terrestrial PTME is associated with a basin-wide interval of rhythmically-bedded laminated reddish-brown and olive-grey siltstone-mudstone couplets[1], a long term negative $\delta^{13}C$ excursion[2,5,13], a change in vegetation and fungal spores[14,15], and significant alterations in community structure and resilience[3,16,17]. Consequently, the changes signify a synchronous, basin-wide restructuring of the terrestrial ecosystem forced by severe environmental degradation[1].

Gastaldo et al.[18] recently obtained a radiometric date of 253.48 ± 0.15 Ma from a silicified ash layer reportedly 60 metres below the palaeontologically defined PTB from Old Lootsberg Pass, Graaff-Reinet District, in the Karoo Basin. These authors claim that this 60 metre interval does not represent enough sedimentation for the biostratigraphically-defined PTB to be synchronous with the marine PTB in China, which is currently dated between 251.880 ± 0.031and 251.941 ± 0.037 Ma[19]. They use their date and the observation of an *in situ* partial dicynodont skull at the base of the Katberg Formation, which they identified as a dicynodontoid, to question the placement of the traditional vertebrate-defined PTB in the Karoo Basin, and instead place the boundary at the base of the Katberg Formation. They also used the presence of typical Permian palynoflora and *Glossopteris*-dominated floral assemblages above their demarkated PTB at Old Lootsberg Pass. There are several problems with these observations and their conclusions in general, but it is beyond the scope of this paper to present a detailed analysis of the interpretations presented by Gastaldo et al.[18].

However, some of their statements do require some comment here as part of the context surrounding our study. We do not consider the radiometric date presented by Gastaldo et al.[18],

nor the dicynodont skull they documented in the Katberg Formation, to be sufficient evidence to change the placement of the currently recognized PTB in the Karoo Basin. The radiometric date supports a latest Permian age (mid-Changxingian) 60 metres below the traditional PTB, which is consistent with the traditional placement of the boundary. As Gastaldo and Neveling[20] note, estimates of sedimentation rates are imprecise due to various assumptions adopted when using modern day sedimentation rates as analogues, as well as factors such as hiatuses and compaction, and so should be treated with caution. A radiometric date closer to the currently positioned PTB would be more appropriate when attempting to correlate the non-marine PTB with the marine record.

In addition to their radiometric date, Gastaldo et al.[18] recovered a partial dicynodont skull from the base of the Katberg Formation that they identified as a Permian "dicynodontoid," and they used this identification to argue against the synchroneity of the extinction fauna given its apparently young stratigraphic position. This argument is invalid on phylogenetic grounds. The dicynodont clade Dicynodontoidea includes lystrosaurid and kannemeyeriiform dicynodonts best known from the Triassic, as well as a paraphyletic assemblage of basal taxa from the Late Permian (i.e., the species previously assigned to the wastebasket taxon *Dicynodon*[21]). Thus, the stratigraphic range of Dicynodontoidea extends from the Late Permian to near the Triassic-Jurassic boundary, when kannemeyeriiform dicynodonts are generally accepted to have become extinct, and a specimen identified only as a dicynodontoid cannot offer any biostratigraphic resolution below this roughly 60 million year time interval. We may assume that Gastaldo et al.[18] are using "dicynodontoid" as a shorthand for "basal dicynodontoid" (i.e., a *Dicynodon*-grade dicynodontoid, not a lystrosaurid or kannemeyeriiform), since this would imply a Late Permian age. If this is the case, their identification is questionable because their published photographs of the specimen do not present any characters that can be used to definitively identify it as a *Dicynodon*-grade dicynodontoid to the exclusion of Lystrosauridae or Kannemeyeriiformes. The specimen could just as easily represent *Lystrosaurus*, which would be completely consistent with an Early Triassic age and the traditional placement of the PTB. Finally, Gastaldo et al.'s[18] treatment of the specimen ignores the possibility of a previously unrecognised survival of a Permian dicynodontoid into the Early Triassic. The Permian *Lystrosaurus* species *L. maccaigi* is known only from the Permian in South Africa[22], but it appears to have survived into the Early Triassic in Antarctica. Therefore, the survival of a Permian dicynodontoid into the Early Triassic would not be completely unprecedented[23,24].

Based on these lines of evidence, we retain the traditional placement of the non-marine PTB in the Karoo Basin. This placement is supported by biostratigraphic research based on hundreds of well-provenanced specimens[1,11], as well as work in other basins (e.g. Europe[25], China[14,15], Russia[26], Australia[27] and Antarctica[28]), and we consider it to represent the terrestrial faunal turnover associated with the PTME. Regardless of the final consensus, the results in our study will remain unaffected by the position of the PTB in the terrestrial realm. The purpose of this study was to examine life history changes during a massive vertebrate faunal turnover, which is represented by the extinction and origin of vertebrates associated with the currently defined PTB in the Karoo Basin.

Growth Marks

Detailed descriptions of the bone histology of the Permo-Triassic therapsids used in this study have been published elsewhere[29-32] and will not be repeated here. However, the occurrence of growth marks in these taxa requires a more detailed explanation. Growth marks include annuli and Lines of Arrested Growth (LAG). Annuli represent a temporary decrease in growth and contain slower forming bone tissues such as lamellar or parallel-fibred bone. They are either avascular or poorly vascularised and have few, flattened osteocyte lacunae. LAGs indicate a temporary, but complete cessation in growth and are represented by a cement line. Experiments

on living taxa have shown that growth marks are deposited annually[33,34]. In Permian therapsids (Supplementary Figs 1-3), cortical bone is punctuated by numerous growth marks (usually three or more) that interrupt the bone tissues prior to the onset of slower-forming bone tissues and is interpreted here as evidence of multi-year growth to somatic and reproductive maturity. Exceptions to this pattern can be found in Triassic therapsids (Supplementary Figs 4-6) where two, but usually one or no growth marks are found before a decrease in growth rate is observed.

The Early Triassic dicynodonts *Myosaurus gracilis*, *Lystrosaurus murrayi*, and *L. declivis* deserve special mention. *Myosaurus* is a tiny emydopoid (BSL_{max}= 46.22 mm) from the *Lystrosaurus* Assemblage Zone (LAZ). A humerus was sectioned from the second largest known *Myosaurus* specimen (BP/1/4269) from South Africa. A larger specimen is known from Antarctica, but we do not consider it here due to possible taxonomic differences with the South African species. As this is the first time that the bone histology of *Myosaurus* has been examined, we describe it in some detail here (Supplementary Fig. 4a). This taxon exhibits fibrolamellar bone with numerous, haphazardly arranged, globular osteocyte lacunae in a woven-fibred bone matrix. The bone tissue is relatively well-vascularised (6.2%) and the vascular canals are arranged in a reticular network. These features indicate high bone deposition rates. The presence of a slight change in bone tissue to slower forming peripheral parallel-fibred bone indicates that this individual was likely a subadult, possibly a late subadult. The lack of secondary remodelling allows an examination of the entire life history of the animal and reveals the absence of growth marks from the cortex, indicating rapid growth to somatic maturity within as little as one year. This is particularly noteworthy as its closest Permian counterparts include *Cistecephalus*, *Dicynodontoides* and *Diictodon*, all of which are relatively small dicynodonts (although larger than *Myosaurus*) and contain numerous annuli or LAGs throughout ontogeny.

The Permian *Lystrosaurus* species reveal fairly extensive remodelling in the inner and mid-cortex, particularly in larger specimens. However, multi-year growth (Fig. 1a and Supplementary Fig. 2b) to maturity at large body size can still be inferred by the number and spacing of growth marks. The Early Triassic *Lystrosaurus murrayi* and *L. declivis* (Supplementary Figs 4b-h) are the most abundant vertebrates known from the LAZ by an order of magnitude. In contrast to Permian *Lystrosaurus* species, they exhibit few growth marks. A single annulus only appears in some individuals from 59% and 63% BSL_{max} in *L. declivis* and *L. murrayi*, respectively. However, the presence of such annuli is not consistent, being present in only one element of an individual, or completely absent, even in some of the larger specimens. One annulus was observed in the largest known specimen of *L. murrayi* and a *L. declivis* specimen that is 82% BSL_{max}. As presence of these annuli are highly variable and generally absent, it was not possible to estimate growth curves for these species. Instead, we superimposed the cross-section from a younger individual containing a growth mark, onto that of the largest for each species. We estimate that only one growth mark is missing from the largest *Lystrosaurus* samples, suggesting that the largest individuals were two years old at the time of death. Although presumably ontogenetically older than the smaller individuals (given their BSL of 185 mm and 213 mm respectively), transitions to slower forming bone tissues and an outer circumferential layer ('OCL') are absent in these largest individuals, indicating that somatic maturity had not been reached. However, resorption cavities extending into the mid-cortex, although less extensive compared to the Permian species, are observed in larger individuals and small scattered secondary osteons are observed throughout the cortex, indicating that they had deviated from the juvenile stage (as secondary remodelling generally increases with age). In order to maintain high abundances in the unstable Early Triassic environment, we propose that *Lystrosaurus* compensated for high mortality rates by reproducing at younger ontogenetic stages compared to their Permian relatives.

BSL Distributions of *Lystrosaurus* and Other Permo-Triassic Dicynodonts

BSL distributions of the two Permian *Lystrosaurus* species were approximately normal. This differed markedly from the two Triassic members of the genus, which had a far lower representation of individuals >70% of the BSL ranges of their respective species (21 and 27% versus 2 and 8% of individuals, respectively; Supplementary Fig. 6). Whereas the Permian species, *L. curvatus* and *L. maccaigi* had similar BSL distributions (X^2=0.295, *df*=1, *p*=0.587), as did the Triassic *L. declivis* and *L. murrayi* (X^2=0.427, *df*=1, *p*=0.513), both Permian taxa differed from both Triassic taxa (X^2=4.023 to 10.879, *df*=1, *p*=0.001 to 0.045). For comparison, we evaluated BSL distributions in another 14 Permian dicynodont taxa for which we had BSL data for a minimum of 13 specimens each (Supplementary Fig. 7). In all cases, except for *Pelanomodon moschops* (*n=12*) and *Dicynodon lacerticeps* (*n*=30), BSL distributions for these taxa were similar to those of the Permian *Lystrosaurus* species, and differed from the Triassic *Lystrosaurus* species in having a higher representation of larger-sized individuals (>70% of the BSL range; Supplementary Table 1). Two taxa (*Oudenodon bainii* and *Rhachiocephalus magnus*) had higher representation of large-bodied individuals compared with other Permian taxa. These results are consistent with results from the bone histology, which indicate lower survival rates in Early Triassic species. The results of *Pelanomodon moschops* and *Dicynodon lacerticeps* is interesting and requires further investigation.

One dicynodont from the Middle Triassic, *Kannemeyeria simocephalus*, showed a BSL distribution similar to that of the Permian, but not Triassic, dicynodonts, hinting at a return to pre-extinction life history strategies. Interestingly, multiple growth marks prior to the shift to slower growth rates are observed in numerous Middle Triassic therapsids (Fig. 2, Supplementary Fig. 4i, 5e, g and Supplementary Appendix 1), supporting the hypothesis that these taxa had returned to a Permian-like life history. However, since the BSL distribution is based on only one taxon (*Kannemeyeria*), with only a few individuals sampled (*n*=12), this hypothesis requires further investigation and a greater sample of Middle Triassic specimens.

Simulated Population Structures, Growth Rates and Extinction Rates

Simulated demographic differences across size classes for two types of species (Type 1 and Type 3 survivorships) are shown in Supplementary Fig. 8. These plots compare predicted size distributions of six potential life history patterns (long- and short-lived, onset of breeding at smaller or larger size classes, and producing higher quantities of offspring, respectively) in a static environment, i.e. assuming zero variability in *K* or in vital rates through time. As expected, survival rates were typically higher for longer-lived than shorter-lived species. However, especially amongst Type 3 populations, a strategy of breeding from a smaller size class (younger age) resulted in lower survival rates – most notably amongst larger size classes. This result is consistent with the trend seen in Triassic *Lystrosaurus*, which had fewer individuals >70% BSL_{max}. Results of matrix model projections for these populations strongly support this interpretation: at stable size distributions, the proportion of individuals in larger size classes was substantially lower in younger-breeding populations than others (Supplementary Fig. 8e and f). These differences are interpreted to be statistically significant based on non-overlapping 95% confidence limits between younger- versus older-breeding populations. The patterns were similar for models in which environmental variability was moderate (±10% of *K*) and high (±50% of *K*).

The outcome of these differences in demographics was that population growth rates were higher amongst simulated populations in which the onset of reproduction was at smaller size classes (in most models 30% versus 50% of the size range; Supplementary Tables 2 and 3).

Although this difference was seldom significant, extinction vortices revealed that younger breeding clearly has an advantage for animals living in variable environments and having curtailed life expectancies. In almost all cases, especially for hypothetical populations with reduced survivorships and life expectancies, extinction rates were lower than in populations which reproduced only later in life, including those with a higher absolute reproductive output (Supplementary Fig. 9; Supplementary Table 2). These scenarios mimic the shorter lifespans and lower relative abundances of larger-sized individuals of *Lystrosaurus* species from the Early Triassic, as inferred from histological and BSL data presented here. Thus, results of these models indicate that onset of reproduction at earlier ages is a life history strategy that was likely favoured in terrestrial vertebrate ecosystems shortly after the extinction event. Faster growth, younger breeding ages, and resultant shortened generation times are all factors that would have played an important role in the survival of *Lystrosaurus* through the end-Permian mass extinction and its dominance in Early Triassic vertebrate communities.

List of Institutional Abbreviations

BP/1/ = Evolutionary Studies Institute (formerly the Bernard Price Institute), University of the Witwatersrand, Johannesburg; NMQR = National Museum, Bloemfontein; RC = Rubidge Collection, Wellwood, Graaff-Reinet; SAM-PK = Iziko Museums of South Africa, Cape Town; UCMP = University of California Museum of Paleontology, Berkeley; UCMZ T = University Museum of Cambridge, Cambridge.

References

1 Smith, R. M. H. & Botha-Brink, J. Anatomy of an extinction: sedimentological and taphonomic evidence for drought-induced die-offs at the Permo-Triassic boundary in the main Karoo Basin, South Africa. *Palaeogeogr. Palaeoclimatol. Palaeoecol.* **396**, 99-118 (2014).

2 Rey, K. *et al.* Global climate perturbations during the Permo-Triassic mass extinctions recorded by continental tetrapods from South Africa. *Gondwana Res.*; DOI:org/10.1016/j.gr.2015.09.008 (2015).

3 Roopnarine, P. D. & Angielczyk, K. D. Community stability and selective extinction during the Permian-Triassic mass extinction. *Science* **350**, 90-93 (2015).

4 Smith, R. M. H. Changing fluvial environments across the Permian-Triassic boundary in the Karoo Basin, South Africa, and possible causes of the extinctions. *Palaeogeogr. Palaeoclimatol. Palaeoecol.* **117**, 81-104 (1995).

5 Ward, P. D., Montgomery, D. R. & Smith, R. M. H. Altered river morphology in South Africa related to the Permian-Triassic Extinction. *Science* **289**, 1740-1743 (2000).

6 MacLeod, K. G., Smith, R. M. H., Koch, P. L. & Ward, P. D. Timing of mammal-like reptile extinctions across the Permian-Triassic boundary in South Africa. *Geology* **28**, 227-230 (2000).

7 Smith, R. M. H. & Ward, P. D. Pattern of vertebrate extinctions across an event bed at the Permian–Triassic boundary in the Karoo Basin of South Africa. *Geol. Soc. Am. Bull.* **29**, 1147-1150 (2001).

8 Tabor, N. J., Montañez, I. P., Steiner, M. B. & Schwindt, D. $\delta^{13}C$ values of carbonate nodules across the Permian–Triassic boundary in the Karoo Supergroup (South Africa) reflect a stinking sulfurous swamp, not atmospheric CO^2. *Palaeogeogr. Palaeoclimatol. Palaeoecol.* **252**, 370-381 (2007).

9 Gastaldo, R. A., Neveling, J., Clark, C. K. & Newbury, S. S. The terrestrial Permian-Triassic boundary event bed is a nonevent. *Geology* **37**, 199-202 (2009).

10 Irmis, R. B. & Whiteside, J. H. Delayed recovery of non-marine tetrapods after the end-Permian mass extinction tracks global carbon cycle. *Proc. R. Soc. Lond. B Biol. Sci.* **279**, 1310-1318 (2012).
11 Viglietti, P. A. *et al.* The *Daptocephalus* Assemblage Zone (Lopingian), South Africa: A proposed biostratigraphy based on a new compilation of stratigraphic ranges. *J. Afr. Earth Sci.* **113**, 153-164 (2016).
12 Anderson, J. M. & Cruikshank, A. R. I. The biostratigraphy of the Permian and Triassic. Pt.5. A review of the classification and distribution of Permo-Triassic tetrapods. *Palaeontol. Afr.* **21**, 15-44 (1978).
13 Ward, P. D. *et al.* Abrupt and gradual extinction among Late Permian land vertebrates in the Karoo Basin, South Africa. *Science* **307**, 709-714 (2005).
14 Vajda, V. & Bercovici, A. The global vegetation pattern across the Cretaceous-Paleogene mass extinction interval: A template for other extinction events. *Global Planet. Change* **122**, 29-49 (2014).
15 Cui, Y. *et al.* Carbon cycle perturbation expressed in terrestrial Permian–Triassic boundary sections in South China. *Global Planet. Change*; DOI:10.1016/j.gloplacha.2015.10.018 (2015).
16 Roopnarine, P. D., Angielczyk, K. D., Wang, S. C. & Hertog, R. Trophic network models explain instability of Early Triassic terrestrial communities. *Proc. R. Soc. Lond. B Biol. Sci.* **274**, 2077-2086 (2007).
17 Roopnarine, P. D. & Angielczyk, K. D. The evolutionary palaeoecology of species and the tragedy of the commons. *Biol. Lett.* DOI:10.1098/rsbl.2011.0662 (2012).
18 Gastaldo, R. A. *et al.* Is the vertebrate-defined Permian-Triassic boundary in the Karoo Basin, South Africa, the terrestrial expression of the end-Permian marine event? *Geology*; DOI:10.1130/G37040.1 (2015).
19 Burgess, S. D., Bowring, S. A. & Shen, S.-z. High-precision timeline for Earth's most severe extinction. *Proc. Natl. Acad. Sci. U S A Biol. Sci.* **111**, 3316-3321 (2014).
20 Gastaldo, R. A. & Neveling, J. The terrestrial Permian-Triassic boundary event is a nonevent: REPLY. *Geology* **40**, e257 (2012).
21 Kammerer, C. F., Angielczyk, K. D. & Fröbisch, J. A comprehensive taxonomic revision of *Dicynodon* (Therapsida, Anomodontia) and its implications for dicynodont phylogeny, biogeography, and biostratigraphy. *J. Vert. Paleontol.* **31**, 1-158; DOI:10.1080/02724634.2011.627074 (2011).
22 Botha, J. & Smith, R. M. H. *Lystrosaurus* species composition across the Permo–Triassic boundary in the Karoo Basin of South Africa. *Lethaia* **40**, 125-137 (2007).
23 Cosgriff, J. W., Hammer, W. R. & Ryan, W. J. The pangaean reptile *Lystrosaurus maccaigi* in the Lower Triassic of Antarctica. *J. Paleo*. **56**, 371-385 (1982).
24 Collinson, J. W., Hammer, W. R., Askin, R. A. & Elliot, D. H. Permian-Triassic boundary in the Transantarctic Mountains, Antarctica. *Geol. Soc. Am. Bull*. **118**, 747-763.
25 Bourquin, S. The Permian–Triassic transition and the onset of Mesozoic sedimentation at the northwestern peri-Tethyan domain scale: Palaeogeographic maps and geodynamic implications. *Palaeogeogr. Palaeoclimatol. Palaeoecol.* **299**, 265-280 (2011).
26 Benton, M. J., Tverdokhlebov, V. P. & Surkov, M. V. Ecosystem remodelling among vertebrates at the Permian-Triassic boundary in Russia. *Nature* **432**, 97-100 (2004).
27 Metcalfe, I., Crowley, J., Nicoll, R. S. & Schmitz, M. High-precision U-Pb CA-TIMS calibration of Middle Permian to Lower Triassic sequences, mass extinction and extreme climate-change in Eastern Australian Gondwana. *Gondwana Res.* **28**, 61-81; DOI:10.1016/j.gr.2014.09.002 (2015).

28 Retallack, G. J. *et al.* The Permian-Triassic boundary in Antarctica. *Antarc. Sci.* **17**, 241-258 (2005).
29 Botha-Brink, J. & Angielczyk, K. D. Do extraordinarily high growth rates in Permo-Triassic dicynodonts (Therapsida, Anomodontia) explain their success before and after the end-Permian extinction? *Zool. J. Linn. Soc.* **160**, 341-365 (2010).
30 Huttenlocker, A. & Botha-Brink, J. Growth patterns and the evolution of bone microstructure in Permo-Triassic therocephalians (Amniota, Therapsida) of South Africa. *PeerJ* **2**, e325 (2014).
31 Botha-Brink, J., Abdala, F. & Chinsamy, A. In *The radiation and osteohistology of non-mammaliaform cynodonts*, The forerunners of mammals: radiation, histology and biology (ed A. Chinsamy) 223-246 (Indiana University Press, 2012).
32 Huttenlocker, A. K. & Botha-Brink, J. Body size and growth patterns in the therocephalian *Moschorhinus kitchingi* (Therapsida: Eutheriodontia) before and after the end-Permian extinction in South Africa. *Paleobiol.* **39**, 253-277 (2013).
33 Francillon-Vieillot, H. *et al.* In *Microstructure and mineralization of vertebrate skeletal tissues*, Skeletal Biomineralization: Patterns, Processes, and Evolutionary Trends Vol. 1 (ed J. G. Carter) 471-530 (Van Nostrand Reinhold, 1990).
34 Hutton, J. M. Age determination of living Nile crocodiles from the cortical stratification of bone. *Copeia* **263**, 31-39 (1986).

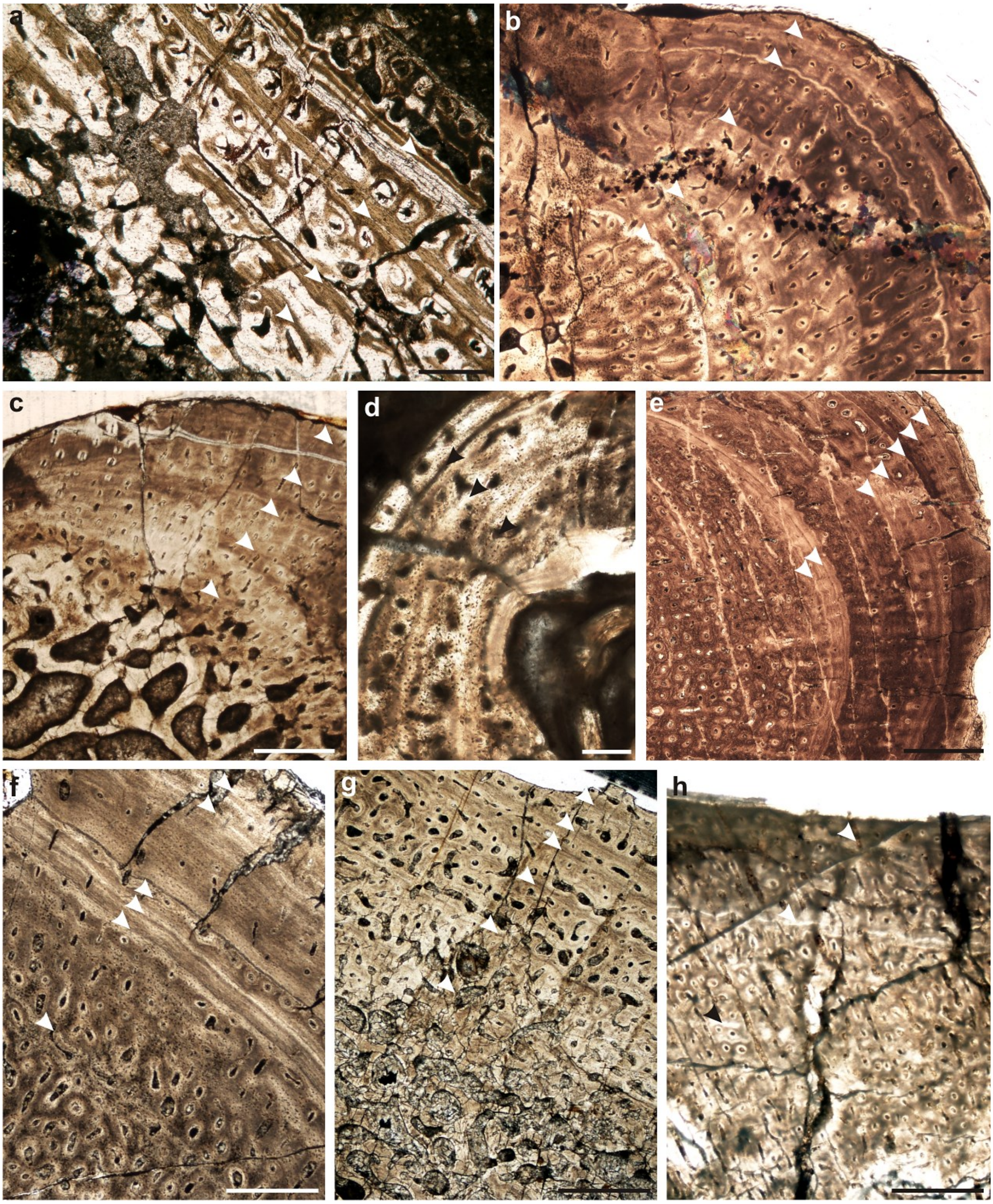

Figure S1. Bone tissue patterns of Permian dicynodont therapsids showing multi-year growth to somatic maturity. (**a**), *Endothiodon*, humerus Sam-pk-k6618. (**b**), *Diictodon*, ulna Sam-pk-k6716a. (**c**), *Dicynodontoides*, tibia NMQR 479a. (**d**), *Cistecephalus*, ulna NMQR 1465b. (**e**), *Oudenodon*, femur Sam-pk-4807. (**f**), *Tropidostoma*, tibia Sam-pk-9960c. (**g**), *Rhachiocephalus*, ulna Sam-pk-3714. (**h**), *Aulacephalodon*, radius Sam-pk-8789. Note that growth marks (arrows) appear during the rapid phase of growth. Scale bars equal 100 µm (**d**); 500 µm (**a-c, f**); 1000 µm (**e, g, h**).

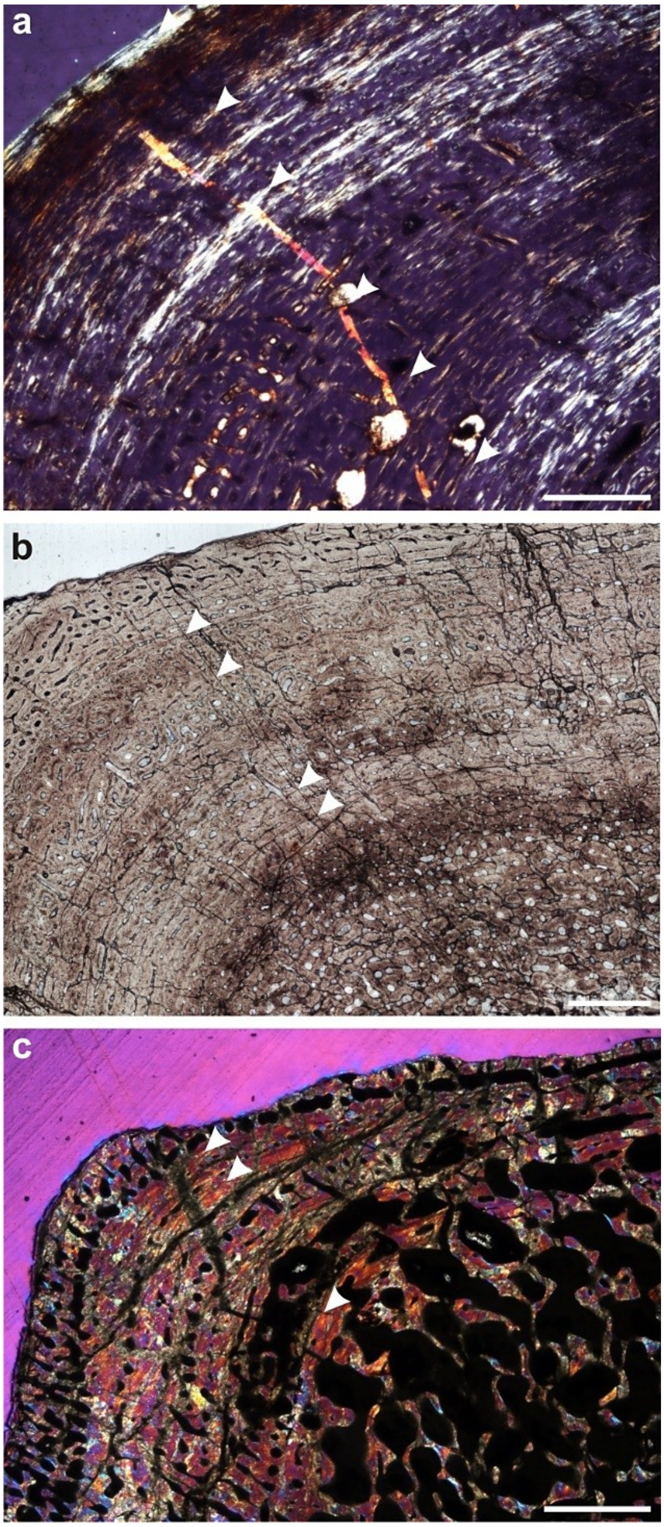

Figure S2. Bone tissue patterns of Permian dicynodont therapsids showing multi-year growth. (a), *Dicynodon,* femur NMQR 3665. (**b**), *Lystrosaurus maccaigi*, tibia NMQR 3689, at 40% BSL_{max}, showing numerous growth marks (arrows) at this young ontogenetic stage. (**c**), *Lystrosaurus curvatus*, humerus NMQR 3651a. Scale bars equal 1000 µm.

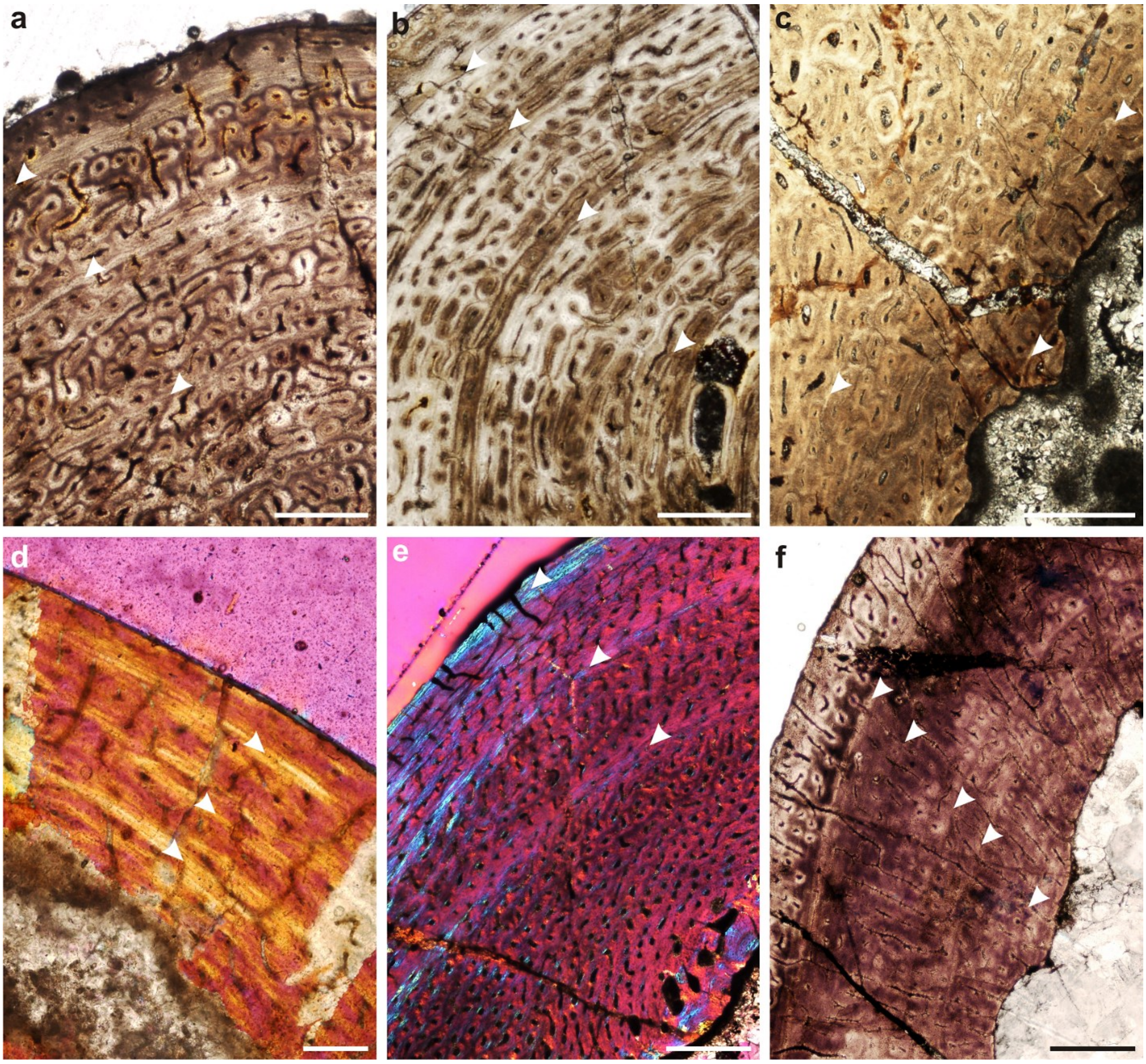

Figure S3. Bone tissue patterns of Permian theriodont therapsids showing multi-year growth to somatic maturity. (**a**), Gorgonopsia indet., femur Sam-pk-10188. (**b**) gorgonopsian *Cyonosaurus,* ulna Sam-pk-k10428b. (**c**) gorgonopsian *Aelurognathus*, humerus Sam-pk-k10000. (**d**), therocephalian *Mirotenthes,* humerus Sam-pk-k6511. (**e**), therocephalian *Ictidosuchoides,* humerus BP/1/4092a. (**f**), therocephalian *Theriognathus,* femur NMQR 3375. Note that growth marks (arrows) appear during the rapid phase of growth. Scale bars equal 100 μm (**d**); 500 μm (**a-c, e, f**).

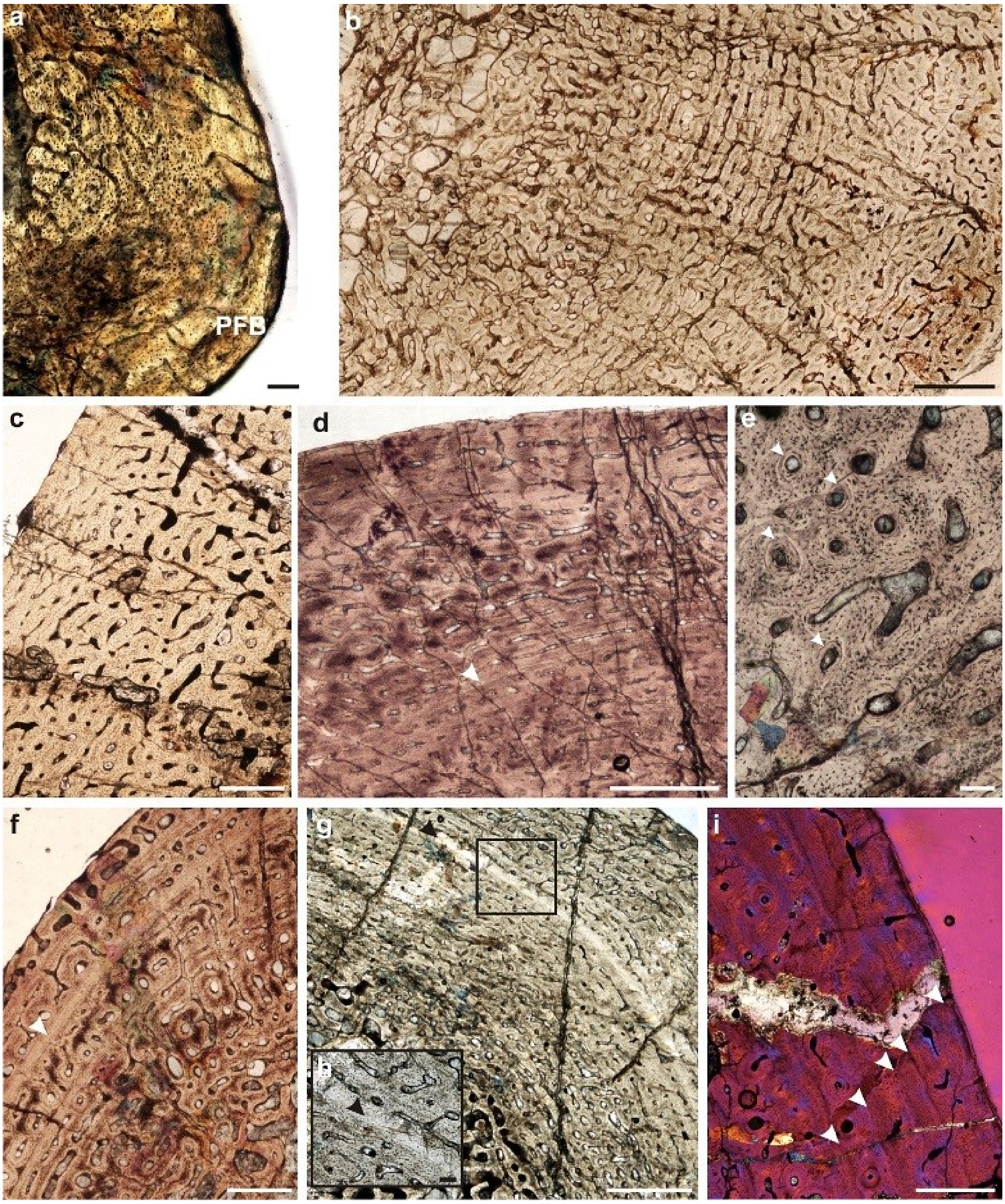

Figure S4. Bone tissue patterns of Early (a-h) and Middle Triassic dicynodont therapsids (i). (**a**), *Myosaurus,* humerus BP/1/4269. (**b**), *Lystrosaurus murrayi*, humerus BP/1/5070. (**c**), *L. murrayi*, femur NMQR 835a. (**d**), *L. murrayi*, humerus BP/1/3236 (100% BSL_{max}), showing a slight decrease in vascularisation towards the periphery and (**e**), higher magnification showing isolated secondary osteons. (**f**) *L. declivis*, tibia NMQR 735b, showing the presence of an annulus at 59% BSL_{max}. (**g**), *L. declivis* and (**h**), higher magnification showing primary tissue interrupted by an annulus in humerus NMQR 1485 at 82% BSL_{max}. (**i**), Middle Triassic dicynodont *Kannemeyeria*, ulna BP/1/3896d. Note the general absence of growth marks in Early Triassic taxa with the Middle Triassic *Kannemeyeria* reverting to the Permian pattern. PFB, parallel-fibred bone. Scale bars equal 100 µm (**a, e, h**); 500 µm (**c, f, i**); 1000 µm (**b, d, g**).

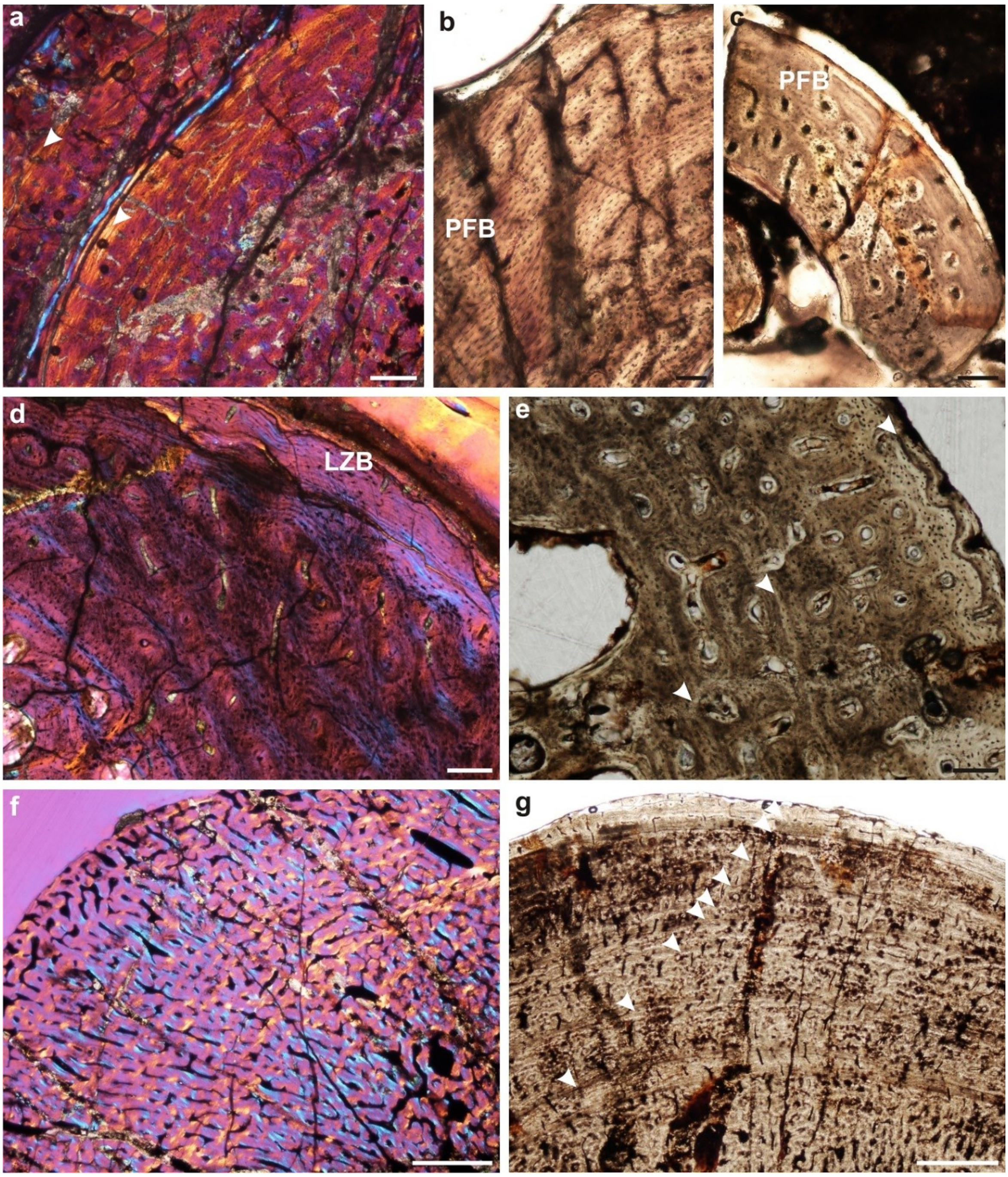

Figure S5. Bone tissue patterns of Early (a-d) and Middle Triassic theriodont therapsids (e-g). (**a**), therocephalian *Olivierosuchus*, humerus NMQR 3605. (**b**), therocephalian *Tetracynodon,* humerus NMQR 3745. (**c**), therocephalian *Scaloposaurus,* humerus Sam-pk-k4638. (**d**), cynodont *Galesaurus,* radius NMQR 3542. (**e**), cynodont *Trirachodon,* tibia Sam-pk-k5881c. (**f**), cynodont *Cynognathus* femur Sam-pk-6235. (**g**), cynodont *Diademodon*, femur NMQR 1208f. Note the general absence of growth marks in Early Triassic taxa with Middle Triassic taxa having reverted to the Permian pattern. PFB, parallel-fibred bone; LZB, lamellar-zonal bone. Scale bars equal 100 µm (**b-e**); 1000 µm (**a, f, g**).

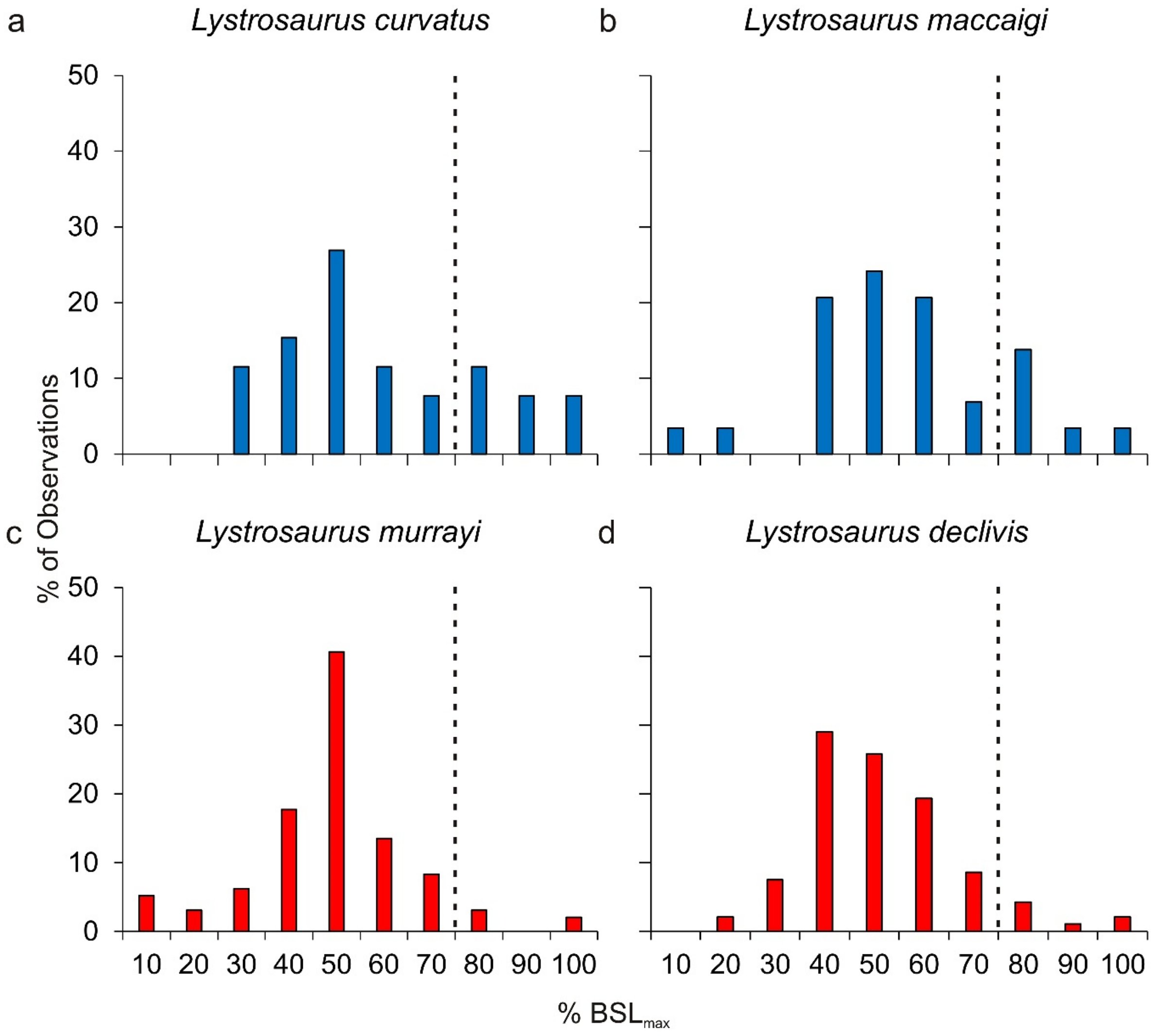

Figure S6. BSL distributions of four *Lystrosaurus* species, showing underrepresentation of individuals from larger size classes in Triassic (red) species compared to Permian (blue) species. Dotted lines: above 70% BSL_{max}; % of BSL_{max}, % of maximum known basal skull length.

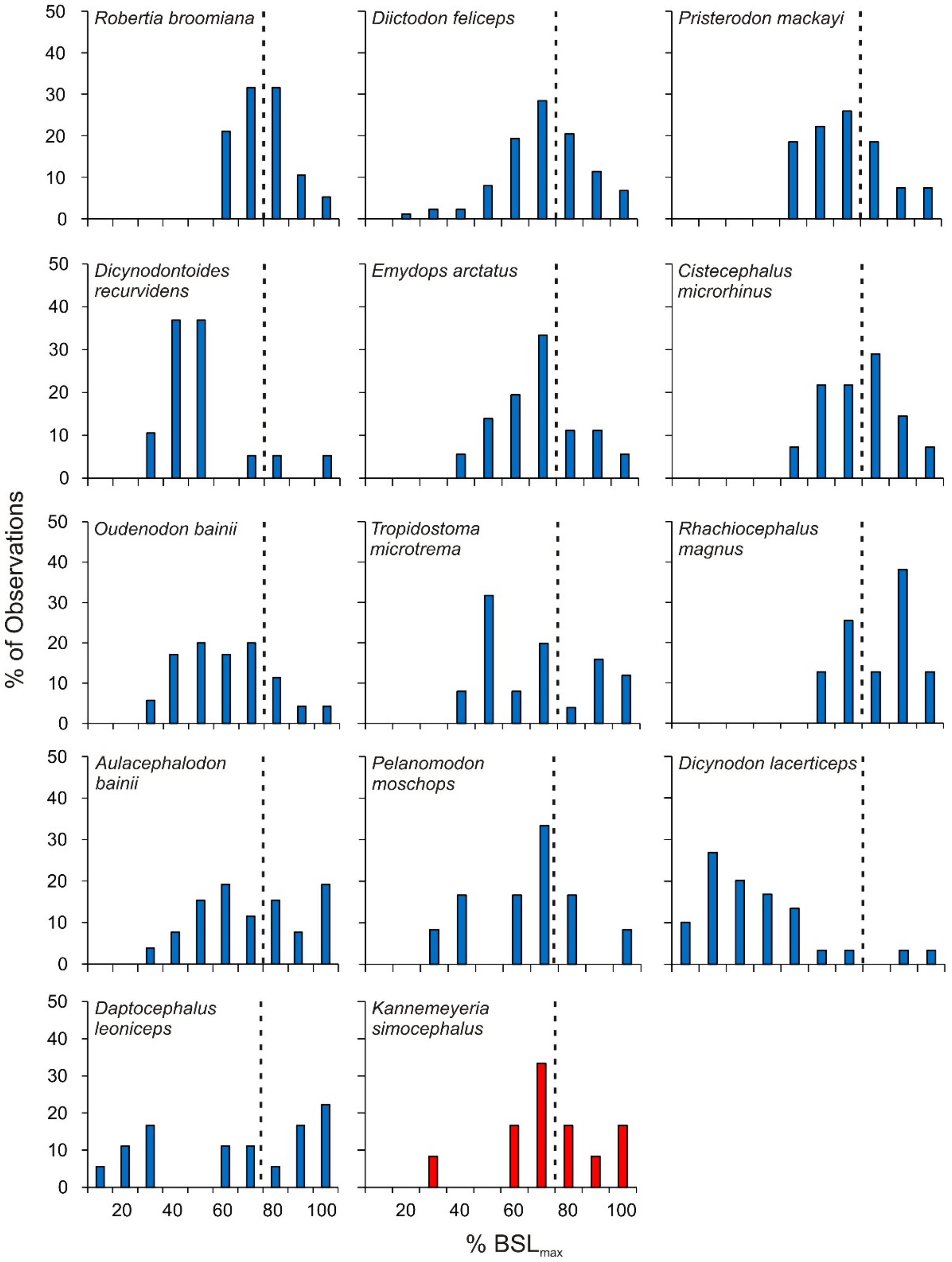

Figure S7. BSL distributions of Permian dicynodonts (blue) and the Middle Triassic *Kannemeyeria simocephalus* (red). The majority differ from the *Lystrosaurus* pattern, in which individuals from this period were more evenly distributed amongst size classes than in the Early Triassic. An exception to this pattern is *D. lacerticeps*. *K. simocephalus* emerged only later, and BSL distributions for this taxon suggest an evolutionary shift back to pre-extinction life history strategies in more stable Middle Triassic environments. Dotted lines: above 70% BSL_{max}.

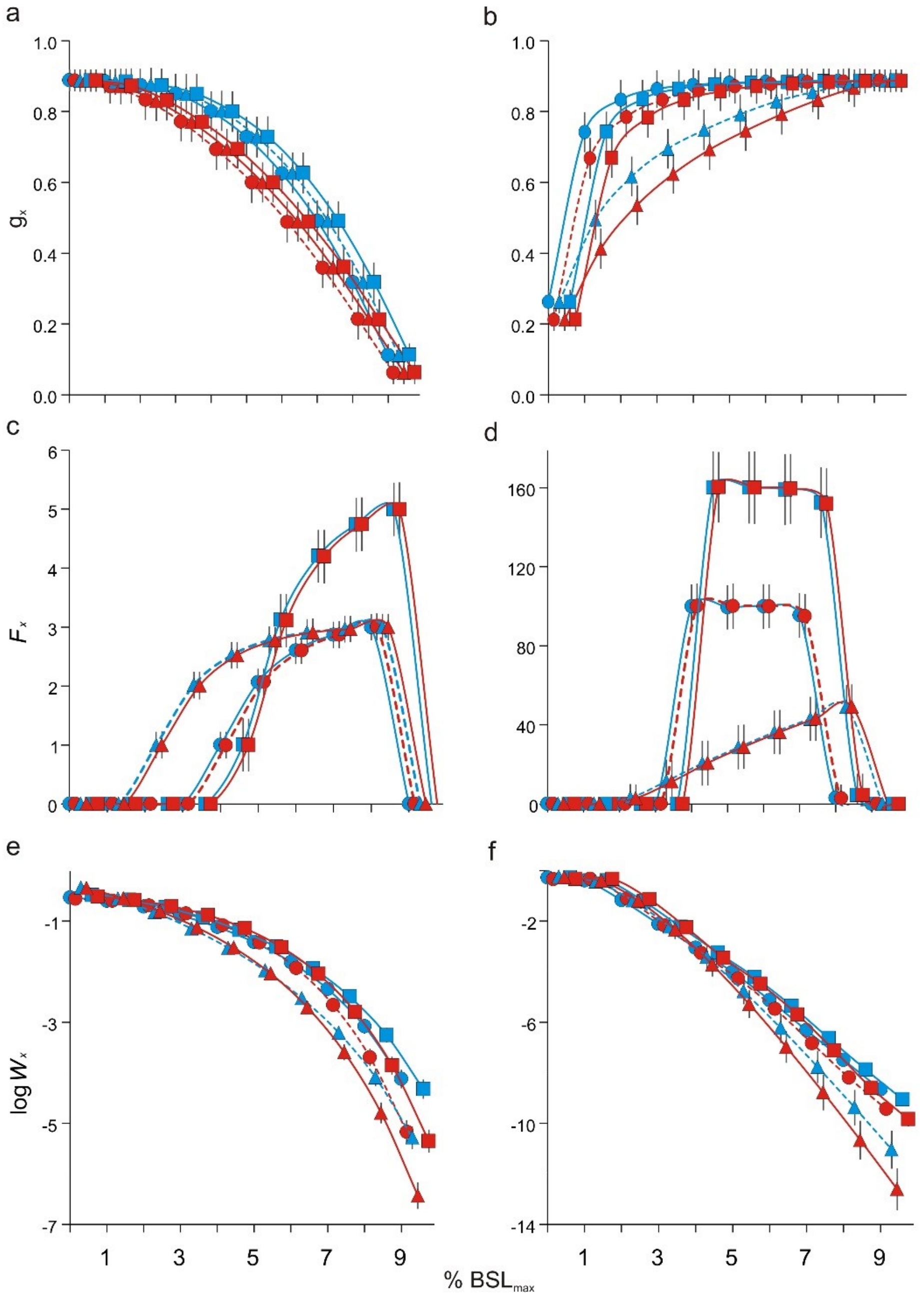

Figure S8. Predicted size class distributions of animals with varying life history strategies, based on hypothetical life table simulations. (a, b) age-specific survivorship schedules; (c, d) fertility schedules; (e, f) stable size distributions derived from matrix model projections. (a, c, e), populations with a Type 1 survivorship; (**b, d, f**), Type 3 survivorships. Symbols are means and error bars depict 95% confidence intervals estimated over 1,000 simulations. For these plots, environmental variability and potential effects on demographic parameters is assumed to be zero. Blue: long life expectancy; Red: short life expectancy. Circles: late breeding, low fecundity; Triangles: early breeding, low fecundity; Squares: late breeding, high fecundity. F_x; fertility of size class x; g_x; age-specific survivorship; log W_x; proportion of individuals in size class x at stable size distributions. % of BSL_{max}, % of maximum known basal skull length.

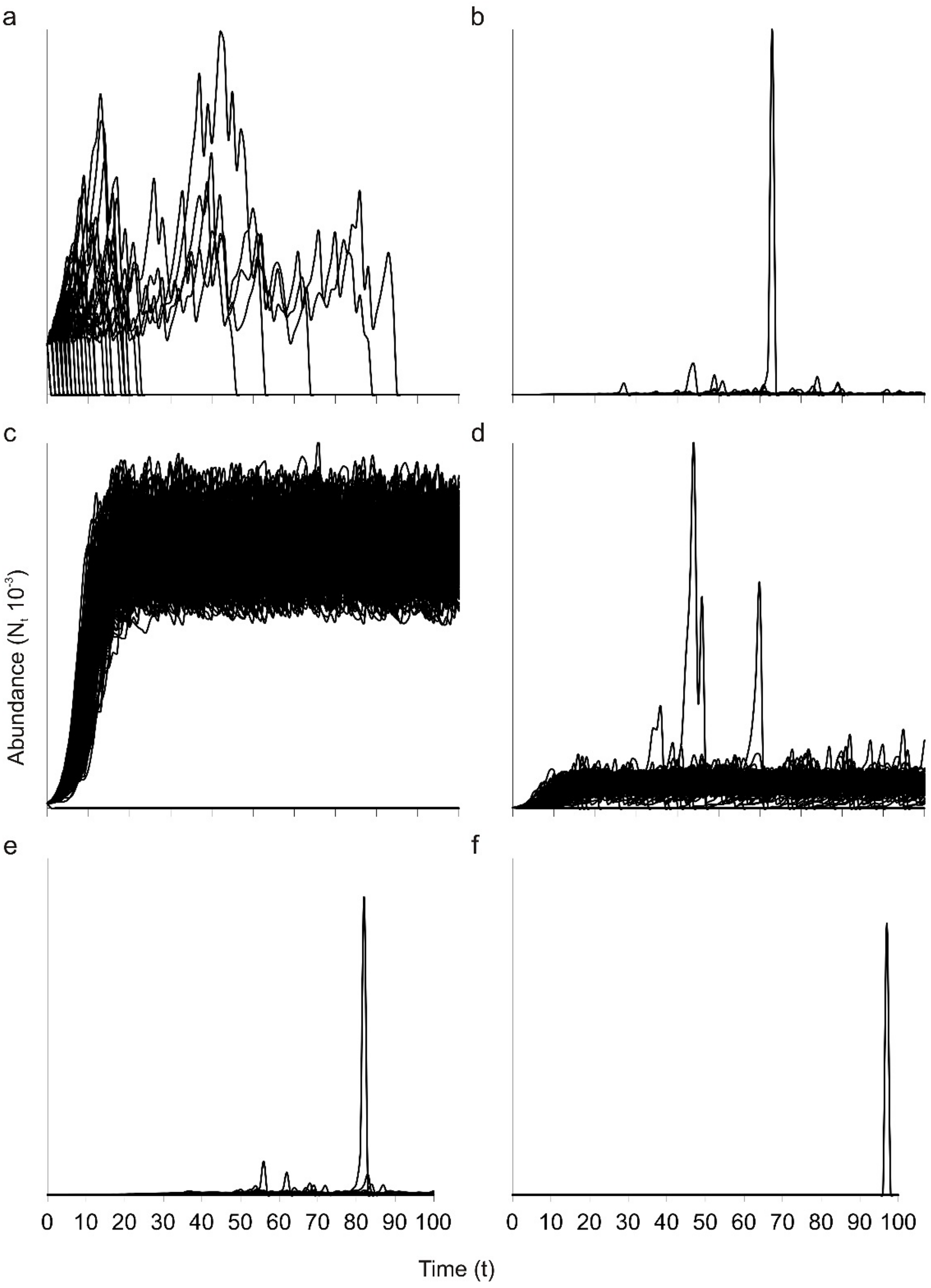

Figure S9. Population growth models (extinction vortices) for hypothetical populations having Type 1 (left hand column) and Type 3 (right) survivorship schedules, all with reduced life expectancies, living in highly variable environments. Abundances on the *y*-axes are plotted over time. Units on the y-axes are not shown as values are arbitrary. For visual clarity, only 200 of the 1 000 simulations are plotted. Note the differences in extinction rates between populations in which the species breed relatively later (**a**, **b**) or earlier (**c**, **d**) in life, and with higher absolute reproductive outputs (**e**, **f**).

Table S1. Comparison of BSL distributions for all Permo-Triassic dicynodont taxa for which sufficient data are available. For each taxon, the frequency of individuals above and below 70% of the body size range is compared with the corresponding frequency distributions observed in all other taxa from the Permian, Early Triassic, and Middle Triassic. Hypothesis tests are chi-squared analyses based on 2x2 contingency tables. Significant differences (p<0.05) are shown in bold.

				**Permian**		**Early Triassic**		**Middle Triassic**	
**Age and Taxon**	***N***	**<=70%**	**>70%**	X^2	p	X^2	p	X^2	p
Permian									
Aulacephalodon bainii	26	57.7	42.3	1.1969	0.2739	27.2853	**0.0000**	0.1000	0.7518
Cistecephalus microrhinus	14	50.0	50.0	1.6628	0.1972	24.3559	**0.0000**	0.0009	0.9758
Daptocephalus leoniceps	18	55.6	44.4	1.0448	0.3067	23.1356	**0.0000**	0.0509	0.8215
**Dicynodon lacerticeps*	30	93.3	6.7	7.5895	**0.0059**	17.8126	**0.0000**	5.2500	**0.0219**
**Dicynodontoides recurvidens*	19	89.5	10.5	2.8870	0.0893	8.9279	**0.0028**	2.4929	0.1144
Diictodon feliceps	88	61.4	38.6	2.7286	0.0986	42.8961	**0.0000**	0.0129	0.9096
Emydops arctatus	36	72.2	27.8	0.0471	0.8281	13.4050	**0.0003**	0.2909	0.5896
Lystrosaurus curvatus	26	73.1	26.9	0.0479	0.8268	9.5907	**0.0020**	0.2846	0.5937
Lystrosaurus maccaigi	29	79.3	20.7	1.0151	0.3137	5.0637	**0.0244**	0.9840	0.3212
Oudenodon bainii	70	80.0	20.0	3.9264	**0.0475**	9.0828	**0.0026**	1.6214	0.2029
**Pelanomodon moschops*	12	75.0	25.0	0.0149	0.9030	0.0009	0.9757	0.1875	0.6650
Pristerodon mackayi	27	66.7	33.3	0.0068	0.9341	16.6455	**0.0000**	0.0193	0.8894
Rhachiocephalus magnus	16	37.5	62.5	6.3703	**0.0116**	42.8639	**0.0000**	0.5056	0.4771
Robertia broomiana	19	52.6	47.4	1.8160	0.1778	27.6436	**0.0000**	0.0036	0.9524
Tropidostoma microtrema	25	68.0	32.0	0.0073	0.9317	14.2530	**0.0002**	0.0436	0.8346
Early Triassic									
Lystrosaurus declivis	93	92.5	7.5	20.0831	**0.0000**	0.1261	0.7225	9.0977	**0.0026**
Lystrosaurus murrayi	96	94.8	5.2	25.4092	**0.0000**	0.1261	0.7225	12.8147	**0.0003**
Middle Triassic									
Kannemeyeria simocephalus	12	58.3	41.7	0.2394	0.6246	13.9028	**0.0002**		

*taxa with <5 individuals in the >70% category; analyses in these cases are based on Yates'-correction for small samples, which is more conservative and may reduce statistical power.

Table S2. Parameter values used for simulating ecological life tables, matrix models to project population growth rates, and population dynamics of Permo-Triassic therapsids approaching body sizes of *Lystrosaurus* spp.

Parameter	Explanation	Type 1	Type 3
$g_x = a + b/x^{\rho}$	age-specific survivorship		
a		1.0	1.0
b		-0.1	-0.1
ρ (long-lived)		-3.0	2.0
ρ (short-lived)		-2.0	0.3
max		0.05	0.05
min		0.90	0.90
$m_x = a - b\rho^x$	fecundity		
a		0.10	0.10
b		3.50	3.50
ρ		0.50	0.50
F_x	fertility		
min		1	1
max (low)		3	20
max (high)		5	30
min breeding age (% of x)			
late		50	50
early		30	30
N_0	initial density in logistic growth model	10	10
K	stable equilibrium density	1000	1000

Table S3. Finite population growth rates (lambda, presented as means with 95% confidence limits in parentheses) and extinction rates (p_{ext}) predicted for populations with different life history characteristics in stable and variable environments. *F*; fecundity; max: maximum; min: minimum.

Model conditions	Minbreeding size	*F*	Type 1 lambda	Type 1 p_{ext}	Type 3 lambda	Type 3 p_{ext}
Stable environment						
Long-lived	Late (50% of max)	Low	1.144 (1.0103 - 1.2778)	0.020	1.4289 (1.1819 - 1.6758)	0.000
	Early (30% of max)	Low	1.6327 (1.5146 - 1.7508)	0.000	1.6531 (0.806 - 2.5002)	0.025
	Late (50% of max)	High	1.2221 (1.0733 - 1.3709)	0.000	1.5178 (1.2077 - 1.8278)	0.000
Short-lived	Late (50% of max)	Low	1.0863 (0.9503 - 1.2224)	0.153	1.1372 (0.8763 - 1.3982)	0.151
	Early (30% of max)	Low	1.6001 (1.4793 - 1.721)	0.000	1.452 (0.6474 - 2.2566)	0.130
	Late (50% of max)	High	1.1532 (1.0047 - 1.3018)	0.021	1.1795 (0.888 - 1.471)	0.095
Variable environment						
Long-lived	Late (50% of max)	Low	1.0179 (0.5179 - 1.5179)	0.384	1.3941 (0.598 - 2.1903)	0.245
	Early (30% of max)	Low	1.4937 (1.0205 - 1.9668)	0.017	1.6454 (0.6904 - 2.6004)	0.063
	Late (50% of max)	High	1.0912 (0.5562 - 1.6262)	0.344	1.4956 (0.5792 - 2.4121)	0.220
Short-lived	Late (50% of max)	Low	0.9718 (0.4629 - 1.4808)	0.451	1.1337 (0.3791 - 1.8883)	0.437
	Early (30% of max)	Low	1.4723 (1.0022 - 1.9424)	0.034	1.4805 (0.5676 - 2.3933)	0.146
	Late (50% of max)	High	1.0436 (0.4998 - 1.5874)	0.387	1.1831 (0.3357 - 2.0305)	0.432
